# Around the Hospitals

**Published:** 1894-03-31

**Authors:** 


### Around the Hospitals.

Notice to the Managers of Institutions.—Special space is now reserved for the insertion of notices of hospital meetings and festivals. The Editor requests that all notices may reach The Hospital Office, 428, Strand, at least one week before the date of each meeting.

**The Perth City and County Infirmary.**—There has been a general increase of work at this infirmary during the past year, and the stay of patients in the wards has also been of longer duration. The income amounted to £2,498, whilst the expenditure exceeded the receipts by £888. The extraordinary expenditure of £2,295 has been devoted to the erection of new laundries and disinfector. A fair amount of legacies have fallen to the lot of the institution. The Perth Infirmary is one of those institutions which receive fever patients from the city and county under special agreement, a system which is stated as working well. During the year 711 in-patients and 2,096 out-patients were treated.

**The Manchester Royal Infirmary.**—The co-operative spirit shown by the institution in affording excellent educational facilities to Owens College, is perhaps the only special feature in its the year's history. The addition of the electric light, now completed, will figure in the present year's report, when its advantages will doubtless be recorded. The Manchester Infirmary is a large and complete corporation. It consists of, besides the general hospital, the convalescent home at Cheadle and the fever hospital at Monsall. The convalescent branch, a most valuable feature in conjunction with curative work for such a population as Manchester, shows unfortunately the least signs of prosperity—a statement which, it is to be hoped, will not be repeated when the next report comes out. The prevalence of small-pox has greatly increased the work at the fever hospital, and the Corporation of Manchester found it necessary at one time to build extra pavilions in connection with it for the reception of patients. At the infirmary 4,451 in-patients were received, and 33,697 out-patients have been treated. The average cost per bed occupied is quoted at £45 16s.

**The Leeds Women and Children's Hospital.**—This, the oldest medical charity in Leeds, tells the tale so common in the experience of the hospital world, that funds have not increased with the large growth of the population; indeed, in this case they show a decrease of £130 in one year. The balance on previous years' accounts have been nearly absorbed by improvements to the hospital, most necessary in the case of an old structure. A new out patients' department has recently been opened, and now attention has to be turned to improved sanitary arrangements. In-patients numbering 269, show a decrease on the admissions of the previous year, whilst the 2,227 out-patients outnumber those of 1892 by 461.

